# Development and evaluation of a replicon particle vaccine expressing the E2 glycoprotein of bovine viral diarrhea virus (BVDV) in cattle

**DOI:** 10.1186/1743-422X-10-35

**Published:** 2013-01-28

**Authors:** John Dustin Loy, Jill Gander, Mark Mogler, Ryan Vander Veen, Julia Ridpath, Delbert Hank Harris, Kurt Kamrud

**Affiliations:** 1 Harrisvaccines, Inc, 1102 Southern Hills Dr. Suite 101, Ames, IA 50010, USA; 2 Department of Animal Science, Iowa State University, Kildee Hall, Ames, IA 50011, USA; 3 United States Department of Agriculture, Agricultural Research Service, Ruminant Diseases and Immunology Research Unit, Ames, IA 50010, USA; 4 Department of Veterinary Diagnostic and Preventive Medicine, Iowa State University, Ames, IA 50011, USA; 5 University of Nebraska-Lincoln, Department of Veterinary and Biomedical Sciences, Lincoln, NE 68502-0907, USA

**Keywords:** BVD, Bovine viral diarrhea virus, Alphavirus replicon, Replicon particle, Vaccine

## Abstract

**Background:**

Bovine viral diarrhea virus is one of the most significant and costly viral pathogens of cattle worldwide. Alphavirus-derived replicon particles have been shown to be safe and highly effective vaccine vectors against a variety of human and veterinary pathogens. Replicon particles are non-propagating, DIVA compatible, and can induce both humoral and cell mediated immune responses. This is the first experiment to demonstrate that Alphavirus-based replicon particles can be utilized in a standard prime/boost vaccination strategy in calves against a commercially significant bovine pathogen.

**Findings:**

Replicon particles that express bovine viral diarrhea virus sub-genotype 1b E2 glycoprotein were generated and expression was confirmed *in vitro* using polyclonal and monoclonal antibodies specific to E2. Vaccine made from particles was generated in Vero cells and administered to BVDV free calves in a prime/boost regimen at two dosage levels. Vaccination resulted in neutralizing antibody titers that cross-neutralized both type 1 and type 2 BVD genotypes following booster vaccination. Additionally, high dose vaccine administration demonstrated some protection from clinical disease and significantly reduced the degree of leukopenia caused by viral infection.

**Conclusions:**

Replicon particle vaccines administered in a prime/boost regimen expressing BVDV E2 glycoprotein can induce cross-neutralizing titers, reduce leukopenia post challenge, and mitigate clinical disease in calves. This strategy holds promise for a safe and effective vaccine to BVDV.

## Findings

### Background

Bovine viral diarrhea virus (BVDV) is an enveloped, positive strand RNA virus in the genus *Pestivirus* (family *Flaviviridae),* and is the causative agent of bovine viral diarrhea (BVD). BVD is one of the most economically significant bovine diseases in the world. Production losses, estimated on a population level, are thought to be $10–40 million US per million calvings [1]. Classically, BVDV has been associated with acute enteric disease; however, BVDV is now understood to be responsible for a broad range of clinical illnesses in cattle including respiratory disease, reproductive loss, and fetal infections [2]. The BVDV E2 gene encodes a 53 kDa major structural glycoprotein that contains a neutralizing epitope that varies among strains [3]. Monoclonal antibodies specific to E2 demonstrate virus neutralizing (VN) ability against both cytopathic and noncytopathic strains of BVDV [4]. Current strategies to reduce losses caused by BVD in infected herds include vaccination with modified live (MLV) or inactivated vaccines and elimination of persistently infected animals. There are no commercially available recombinant or vectored vaccines, and thus producers are limited to either modified live MLV or inactivated vaccine approaches [5]. Additionally, some success has been shown experimentally using BVD pseudovirions, which have a deletion in the structural genes, and are rescued using homologous helper RNA in *trans*[6].

A replicon-based expression system has been derived from the alphavirus (family *Togaviridae*) Venezuelan Equine Encephalitis (VEE) virus [7,8]. Alphavirus-based replicon particles have numerous advantages for vaccine development including; accurate production of native proteins and a propagation-defective nature that makes the system only capable of a single infection cycle [9]. This viral vector has previously been used to express genes from numerous pathogens including human strains of influenza virus, simian immunodeficiency virus, Norwalk virus, Ebola virus, smallpox virus, Lassa virus, and equine arteritis virus [10-16]. RP have a robust safety profile. It has been demonstrated that RP are not shed from vaccinated animals and as such cannot spread to unvaccinated animals. Furthermore, RP have been tested for the presence of replication competent virus that may arise by recombination during production of RP and have been shown to lack the ability to revert to virulence [17]. Alphavirus RP have also been tested in cattle against foot and mouth disease virus (FMDV) (Kurt Kamrud, personal communication) but have not been tested against a commercially-significant cattle disease in the United States using a prime/boost vaccination strategy. Therefore, it is proposed that alphavirus RP that express the E2 glycoprotein from BVDV would provide a novel, safe, and effective approach to control BVD.

### Methods

**Figure 1:**
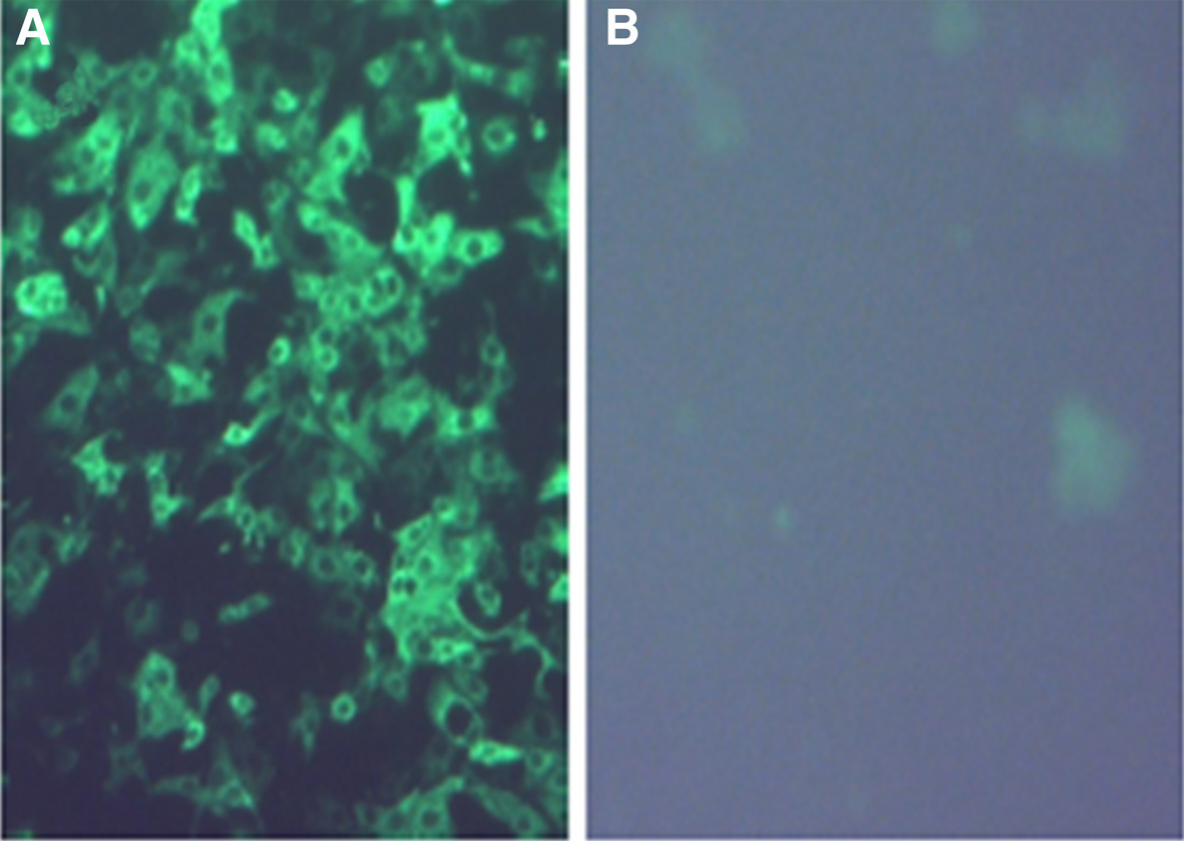
**Indirect immunofluorescence assay of replicon particle** (**RP**) **infected Vero cells.** Vero cell monolayers were infected with RP expressing either (**A**) the E2 glycoprotein gene of BVDV subgenotype 1b strain NY1 or (**B**) a non-BVDV-derived gene as a control. Cells were fixed and subsequently stained with a monoclonal antibody specific to BVDV 1b E2 and a fluorescent-conjugated anti-mouse secondary antibody.

The E2 glycoprotein gene was *de novo* synthesized (DNA2.0) using sequence from BVDV subgenotype 1b strain NY1 (Genbank: AY027671). The E2 gene was cloned into a replicon vector plasmid as previously described [8] and the sequence was confirmed to ensure no mutations were introduced in the cloning process. RNA was generated by *in vitro* transcription of linearized replicon plasmid DNA using T7 RNA polymerase as described previously [8]. RP were generated by co-electroporation of E2 replicon RNA and structural gene helper RNAs into Vero cells and subsequent harvest of the particles [16]. Vero cell monolayers were then infected with E2 RP at an MOI of 10 and the presence of E2 expression was confirmed by western blot analysis of E2 RP infected cell lysates using hyperimmune polyclonal swine serum (data not shown). In addition, E2 protein expression was confirmed by indirect immunofluorescence assay using E2 specific monoclonal antibodies (Figure 1).

Seven (7) calves, 8 weeks of age, were sourced from a BVD free herd. Each animal was tested and found negative for BVDV antibodies by viral neutralization assay (VN), BVDV antigen in earnotch samples (BVD immunohistochemistry), and were negative for circulating virus in whole blood by PCR. Three (3) calves were randomly assigned to each of 2 blocks (experimental groups) and one (1) calf was assigned to a single block (placebo). The calves were acclimated for 7 days before onset of the trial. Calves were injected intramuscularly (IM) with 2 mL of 5 × 10^6^ infectious units (IU)/mL RP (1 × 10^7 ^IU total), 5 × 10^5 ^IU/mL RP (1 × 10^6 ^IU total), or a placebo control of RP diluent (1% normal bovine serum, 5% sucrose in PBS). Personnel sampling and scoring the animals were blinded to the treatment groups.

Serum was collected from calves on days 0, 21, 28, 35, 42, 49, and on 56. Whole blood was collected in tubes containing EDTA 1 day prior to challenge through 14 days post challenge (a total of 16 days). On day 42 animals were challenged with 1×10^6 ^TCID50/mL of BVD1b strain NY1 intranasally (USDA distributed efficacy challenge material). Neutralizing antibody responses were assessed using representative viruses from Type 1 and Type II BVDV strains (Singer and 296c, respectively) using day 0, 21, 28, and 42 serum samples. Clinical disease was scored on a scale from 0–3 each day. White blood cell (WBC) counts were assessed on whole blood samples collected in tubes containing EDTA using an automated cell counter (Iowa State University, Veterinary Pathology). The degree of leukopenia following virus challenge was assessed by subtracting the maximum reduction in circulating WBCs from the baseline level (calculated from a mean of two samples prior to challenge).

### Results

**Figure 2:**
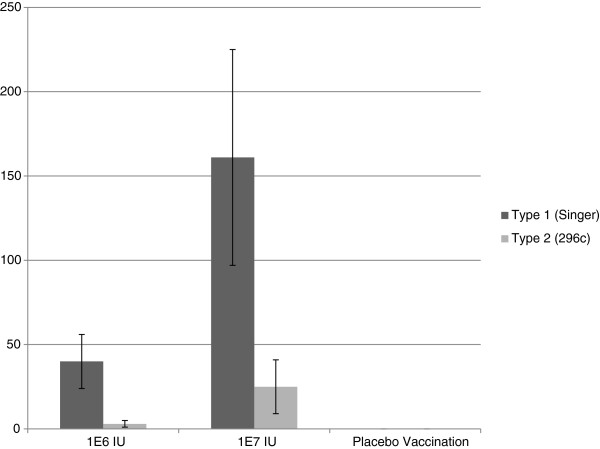
**Geometric mean of viral neutralizing antibody dilutions on day of virus challenge.** The 1×10^7 ^IU vaccinated group had significantly higher geometric mean VN titers (P=.04 by oneway ANOVA) than the 1×10^6^ IU dosage level. Reference strains used were Singer (type 1) and 296c (type 2).

**Figure 3:**
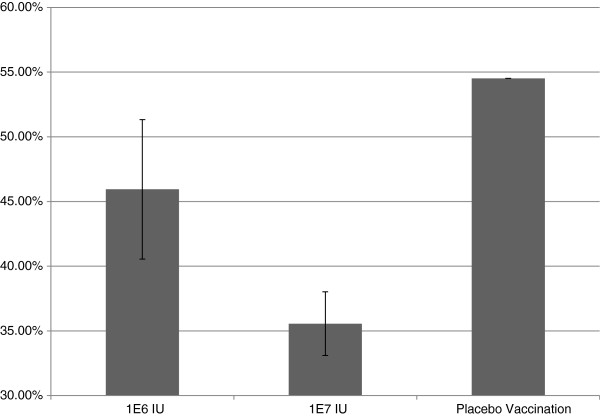
**Y**-**axis is percent depletion of circulating white blood cells when compared to pre**-**challenge baseline (set to 100%).** The highest levels of depletion were seen in the placebo groups with a dose dependent decrease in WBC reduction in vaccinated groups. Error bars indicate standard error from mean percentage depletion.

**Figure 4:**
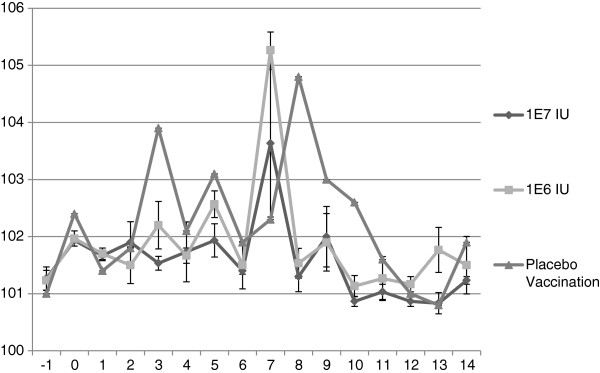
**Mean rectal temperatures** (°**F**) **post challenge with BVD1b NY1** (**Y**-**axis.**) X-axis is days post viral challenge. Error bars indicate standard error from mean.

**Figure 5:**
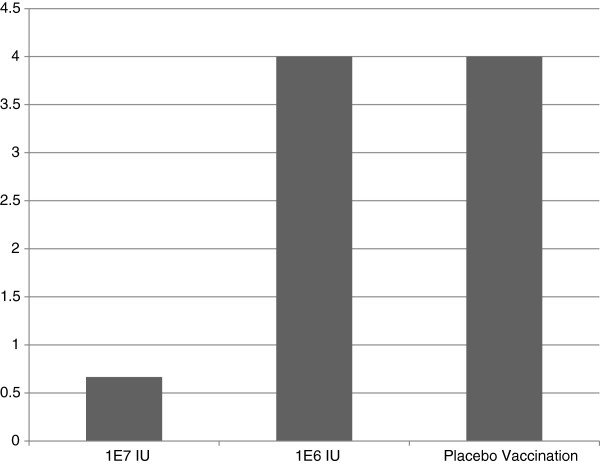
**Mean clinical scores over the course of the study.** Scores were based on presence of mild nasal discharge, mild ocular discharge, or mild depression. No differences were seen in clinical outcomes between low dose E2 RP and placebo controls in clinical disease.

Serum from the control animal was VN negative on the day of challenge. A dose dependent heterologous VN response was observed in animals to sub-genotypes BVD1a and BVD2. VN titers were induced in all vaccinated animals to Type 1 strains and most animals to Type 2 strains in the high dose group. Significant differences by one-way ANOVA were observed in VN geometric mean titers between low and high dose E2 RP vaccinated groups (Figure 2). The serum of all E2 RP vaccinated animals was positive at termination by western immunoblotting against recombinant E2 glycoprotein. Both E2 RP treated groups showed a reduced degree of leukopenia when compared with the control; the high dose E2 RP dose having a greater impact on reducing leukopenia (Figure 3). Febrile responses were noted in all animals at some study timepoint following virus challenge (Figure 4). The placebo group demonstrated a febrile response before E2 RP vaccinated animals at 3 days post challenge (dpc) (Figure 4). All experimental groups demonstrated febrile responses from days 7–9 post challenge with a maximum of 105.8°F (Figure 4). Temperatures returned to normal levels in all E2 RP vaccinated animals by day 9 post challenge; however, temperatures remained elevated for several additional days in the control animal (Figure 4). Clinical disease scores (nasal/ocular discharge and/or depression) were collected for all animals. Protection from clinical disease after challenge was noted in 2 of the 3 high dose E2 RP vaccinated animals. The clinical disease scores for the low dose E2 RP group and placebo control were not different (mean clinical score = 4); In contrast, the high dose E2 RP group demonstrated significantly reduced disease severity and degree of clinical signs (mean clinical score = 0.66) (Figure 5).

In summary, these data demonstrate that RP expressing BVD1b NY1 E2 glycoprotein can induce dose dependent immune responses specific to BVDV. Importantly, the E2 RP induce a cross-reactive VN antibody response to Type 1a and Type 2 BVD strains. This response was significantly higher in animals that received the 1 × 10^7 ^IU E2 RP dosage level. Furthermore, the vaccine impacted clinical parameters post-challenge with a homologous BVDV strain and reduced the degree of leukopenia post-challenge when compared to the control. The current study does not address if the heterologous neutralization titers indicate efficacy against heterologous challenge, however, this will be addressed in future experiments. We believe that BVDV E2 RP represent an attractive alternative to MLV or inactivated vaccine approaches because of the efficacy demonstrated here, the safety profile of the vector, and because E2 RP may allow differentiation of infected and vaccinated animals capable of supporting BVDV eradication programs.

The use of biohazardous materials and the calf experimental protocol were reviewed and approved by the ISU Institutional Biosafety Committee (IBC) and the Institutional Animal Care and Use Committee (9-11-7225-B).

## Competing interests

The authors declare that they have no competing interests.

## Authors’ contributions

JDL, KIK, and DLH conceived the study. JDL, MAM, JRG, RVV and KIK developed, manufactured, and tested the vaccine. JR performed assays and provided guidance. JDL wrote the manuscript. All authors read and approved the manuscript.
